# Gender-Specific Effects of Genetic Variants within Th1 and Th17 Cell-Mediated Immune Response Genes on the Risk of Developing Rheumatoid Arthritis

**DOI:** 10.1371/journal.pone.0072732

**Published:** 2013-08-30

**Authors:** Rafael Cáliz, Luz María Canet, Carmen Belén Lupiañez, Helena Canhão, Alejandro Escudero, Ileana Filipescu, Juana Segura-Catena, María José Soto-Pino, Manuela Expósito-Ruiz, Miguel Ángel Ferrer, Antonio García, Lurdes Romani, Alfonso González-Utrilla, Teresa Vallejo, Eva Pérez-Pampin, Kari Hemminki, Asta Försti, Eduardo Collantes, João Eurico Fonseca, Juan Sainz

**Affiliations:** 1 Rheumatology Department, Virgen de las Nieves University Hospital, Granada, Spain; 2 Genomic Oncology Area, GENYO, Centre for Genomics and Oncological Research: Pfizer/University of Granada/Andalusian Regional Government, Granada, Spain; 3 Rheumatology Research Unit, Instituto de Medicina Molecular, Faculdade de Medicina da Universidade de Lisboa, Lisbon, Portugal; 4 Rheumatology Department, Santa Maria Hospital–CHLN, Lisbon, Portugal; 5 Rheumatology Department, Reina Sofía Hospital, Córdoba, Spain; 6 FIBAO Fundation, Virgen de las Nieves University Hospital, Granada, Spain; 7 Rheumtology Unit, Instituto de Investigacion Sanitaria-Hospital Clinico Universitario de Santiago, Santiago de Compostela, Spain; 8 Division of Molecular Genetic Epidemiology, German Cancer Research Center, Heidelberg, Germany; 9 Center for Primary Health Care Research, Clinical Research Center, Malmö, Sweden; Kunming Institute of Zoology, Chinese Academy of Sciences, China

## Abstract

The present study was conducted to explore whether single nucleotide polymorphisms (SNPs) in Th1 and Th17 cell-mediated immune response genes differentially influence the risk of rheumatoid arthritis (RA) in women and men. In phase one, 27 functional/tagging polymorphisms in C-type lectins and MCP-1/CCR2 axis were genotyped in 458 RA patients and 512 controls. Carriers of *Dectin-2*_rs4264222T_ allele had an increased risk of RA (OR = 1.47, 95%CI 1.10–1.96) whereas patients harboring the *DC-SIGN*_rs4804803G_, *MCP-1*_rs1024611G_, *MCP-1*_rs13900T_ and *MCP-1*_rs4586C_ alleles had a decreased risk of developing the disease (OR = 0.66, 95%CI 0.49–0.88; OR = 0.66, 95%CI 0.50–0.89; OR = 0.73, 95%CI 0.55–0.97 and OR = 0.68, 95%CI 0.51–0.91). Interestingly, significant gender-specific differences were observed for *Dectin-2*_rs4264222_ and *Dectin-2*_rs7134303_: women carrying the *Dectin-2*_rs4264222T_ and *Dectin-2*_rs7134303G_ alleles had an increased risk of RA (OR = 1.93, 95%CI 1.34–2.79 and OR = 1.90, 95%CI 1.29–2.80). Also five other SNPs showed significant associations only with one gender: women carrying the *MCP-1*_rs1024611G_, *MCP-1*_rs13900T_ and *MCP-1*_rs4586C_ alleles had a decreased risk of RA (OR = 0.61, 95%CI 0.43–0.87; OR = 0.67, 95%CI 0.47–0.95 and OR = 0.60, 95%CI 0.42–0.86). In men, carriers of the *DC-SIGN*_rs2287886A_ allele had an increased risk of RA (OR = 1.70, 95%CI 1.03–2.78), whereas carriers of the *DC-SIGN*_rs4804803G_ had a decreased risk of developing the disease (OR = 0.53, 95%CI 0.32–0.89). In phase 2, we genotyped these SNPs in 754 RA patients and 519 controls, leading to consistent gender-specific associations for *Dectin-2*_rs4264222_, *MCP-1*_rs1024611_, *MCP-1*_rs13900_ and *DC-SIGN*_rs4804803_ polymorphisms in the pooled sample (OR = 1.38, 95%CI 1.08–1.77; OR = 0.74, 95%CI 0.58–0.94; OR = 0.76, 95%CI 0.59–0.97 and OR = 0.56, 95%CI 0.34–0.93). SNP-SNP interaction analysis of significant SNPs also showed a significant two-locus interaction model in women that was not seen in men. This model consisted of *Dectin-2*_rs4264222_ and *Dectin-2*_rs7134303_ SNPs and suggested a synergistic effect between the variants. These findings suggest that *Dectin-2*, *MCP-1* and *DC-SIGN* polymorphisms may, at least in part, account for gender-associated differences in susceptibility to RA.

## Introduction

Rheumatoid arthritis (RA) is an autoimmune disease characterized by chronic inflammatory activity in the synovial joints often leading to progressive cartilage and bone destruction [1]. Although etiology of RA is still unknown, it has been suggested that activated macrophages and dendritic cells (DCs), rather than T-cells, may be important for the onset of the disease. In the RA synovium, macrophages mature into tissue macrophages and differentiate into DCs leading to massive release of a wide number of pro-inflammatory mediators [2]–[4] and to the induction of adhesion (C-type lectins) and co-stimulatory molecules (CD40, CD80, CD86) that participate in the activated macrophage- and DC-induced T-cell proliferation [5], [6]. Among all these immune mediators, *MCP-1/CCL2*, a chemokine highly expressed in the synovial fluid of RA patients [7], binds to CCR2 and promotes the recruitment of antigen-presenting cells and T cells [8] whereas C-type lectins such as *Dectin-1*, *Dectin-2* and *DC-SIGN* regulate the monocyte-induced T-cell activation in the RA synovium [5], [6]. C-type lectins also modulate adaptive immune responses, of which especially Th17 responses are implicated in the stimulation of osteoclastogenesis and bone destruction during RA [9].

RA is three times more frequent in women than men [10] and emerging data also suggest that women are more likely to present a worse course of the disease and to become severely disabled [11], [12]. Although different hypotheses concerning sex-related differences in RA incidence and severity have been generated, the hypothesis suggesting a sexual dimorphism in the intensity of immune responses remains as one of the most probable mechanisms in promoting and establishing a different synovial membrane inflammation and, subsequently, different levels of cartilage and bone erosion [13], [14]. Women have higher immunoglobulin levels than men, they show stronger Th1-type cell-mediated immune responses and they have higher absolute numbers of CD4^+^ lymphocytes and a more proactive cytokine profile [15], [16], which likely contribute to their increased autoimmune responses [17]. Experimental studies in rodents have also shown different immune responses between female and male animals and an equivalent sexual dimorphism in the incidence of RA [18]. The underlying reasons for this gender bias are still unknown, but studies in monozygotic twins have suggested that genetic factors may, at least in part, account for sex-related differences in the immune responses [19] and, consequently, in the susceptibility to autoimmune diseases. Genetic factors implicated in RA have been widely studied using both candidate genes [20] and whole-genome screens [21] but, so far, only a few studies have investigated the link between SNPs and the gender-associated differences in susceptibility to RA [22], [23]. Considering these facts, the present study was designed to evaluate the influence of 27 tagging and potentially functional SNPs within the *MCP-1*, *CCR2*, *DC-SIGN*, *Dectin-1*, and *Dectin-2* genes in the risk to develop RA in women and men, separately.

## Materials and Methods

### Study Population

In phase 1, all participants enrolled were Caucasian and recruited at the department of Rheumatology of the Virgen de las Nieves (Granada, Spain) and Reina Sofia (Córdoba, Spain) hospitals. All participants gave their written informed consent to participate in the study, which was approved by the ethical review committee of participant institutions (Virgen de las Nieves University Hospital, Granada, Spain; Reina Sofia Hospital, Córdoba, Spain). The study was performed according to the principles of the Declaration of Helsinki. The population consisted of 970 participants, 458 RA patients (360 women and 98 men) and 512 healthy controls (217 women and 295 men). Rheumatoid patients were treated at their respective departments of Rheumatology from January 2004 to January 2010. The diagnosis of RA was assigned by physician investigators and fulfilled the 1987 American College of Rheumatology (ACR) criteria. We chose DAS28 as a measure of disease activity as it is a validated score for established RA. Moderate to high activity disease was defined as DAS28≥3.2 while low disease activity was defined as DAS28<3.2. Controls were blood donor subjects randomly recruited at the Regional Blood Transfusion and Tissue Centre (Granada-Almería, Spain).

In phase 2, in order to increase the statistical power of the study and confirm both overall and gender-specific associations, we extended the study by recruiting additional RA cases (n = 831) and controls (n = 550) from our own institution as well as from other collaborating institutions. Seven hundred and seventy-three RA patients and 201 controls were recruited from the Santa Maria Hospital–CHLN (Lisbon, Portugal). Fifty-eight additional RA patients were recruited from the University Clinical Hospital of Santiago de Compostela (Santiago de Compostela, Spain). We also recruited 349 healthy controls from our own institution (Virgen de las Nieves University hospital, Granada, Spain; n = 260) as well as from the Reina Sofía hospital (Córdoba, Spain; n = 89). All participants gave their written informed consent to participate in the study, which was approved by the respective ethical review committee of participant institutions. Thirty-four non-Caucasian subjects were excluded from the statistical analyses (n = 34) and some additional patients and controls were also removed for technical reasons (low DNA quality, unknown age or gender, etc.). Seven hundred and fifty-four RA patients (626 women and 128 men) and 519 healthy controls (348 women and 171 men) were finally available for genotyping.

### SNP Selection and Genotyping

Twenty-seven tagging/functional SNPs within *DC-SIGN*, *Dectin-1*, *Dectin-2*, *MCP-1* and *CCR2* were selected to genotype the entire panel of individuals (Table 1; Supplementary material). The aim of the SNP tagging was to identify a set of SNPs that efficiently tags all the known SNPs while the functional approach was used to determine the net impact of potentially functional variants within *DC-SIGN, Dectin-1, Dectin-2, MCP-1 and CCR2* genes on RA risk. Tagging SNPs were selected using Haploview Tagger program (http://www.broad.mit.edu/mpg/haploview/; http://www.broad.mit.edu/mpg/tagger/). SNPs with a MAF>0.05 were included in the selection of tag SNPs using a pairwise tagging with a minimum r^2^ of 0.8. In this selection we forced the inclusion of the *DC-SIGN*_rs4804803_, *MCP-1*_rs1024610_ and *MCP-1*_rs1024611_ polymorphisms as their functionality has been suggested [24]–[26]. One extra SNP within DC-SIGN (rs11465384) and in strong linkage disequilibrium (LD) with at least 4 SNPs was selected as redundant SNP in case of genotyping failure. Genomic DNA was extracted from peripheral blood mononuclear cells (PBMCs) using Qiagen Mini Kit (Qiagen, Valencia, CA, USA). Genotyping of *DC-SIGN*, *Dectin-1*, *Dectin-2*, *MCP-1* and *CCR2* polymorphisms was carried out using allele-specific KASPar® assays (LGC Genomics KBioscience, London, United Kingdom) in a 384-well plate format (Applied Biosystems, Foster City, CA, USA) where population samples (RA and healthy controls) were randomly distributed. KASPar reactions were performed using KASPar assay mix (containing probes) and KASPar kit containing 2X Reaction Mix and MgCl2 (50 mM). Touch-down PCR conditions were: denaturation at 94°C for 15 min, 10 cycles of denaturation at 94°C for 20 sec, annealing at 61°C for 60 sec (dropping –0.6°C per cycle) and 26 cycles of denaturation at 94°C for 10 sec, annealing at 55°C for 60 sec. Recycling conditions were 94°C for 10 sec, annealing and elongation at 60°C for 60 sec. PCR products were analyzed with the ABI Prism 7900HT detection system using the SDS 2.4 software (Applied Biosystems). For internal quality control, 5% of samples were randomly selected and included as duplicate. Concordance between the original and the duplicate samples for the 27 SNPs analyzed was ≥99.0%. Call rates for all SNPs were ≥95.0% with the exception of the *Dectin-1*_rs11053599_ SNP with a call rate <90.0%. This latter SNP was excluded from further analysis.

**Table 1:** Selected SNPs within DC-SIGN, Dectin-1, Dectin-2, MCP-1 and CCR2 genes.

Gene andSNP position	dbSNPrs#	Location	Aachange	Nucleotide substitution	MAF ‡	Hypothetical function and/orreported associations relatedto autoimmune and/orinfection disorders	References*
DC-SIGN_c.-139	rs2287886	Promoter	–	A/G	0.32	Affects transcriptional activity and DC-SIGN mRNA expression level; associated with protectionagainst IPA infection; associated with several infection and immune-related diseases suchas hCMV, HIV-1, Dengue, TB, parenteral infection, SARS.	[1]–[5]
DC-SIGN_c.-336	rs4804803	Promoter	–	A/G	0.24	Affects transcriptional activity and DC-SIGN mRNA expression; associated with risk ofparenteral infection, HIV-1, HCV, dengue and tuberculosis.	[5]–[11]
DC-SIGN_c.2797	rs4804800	3′-UTR	–	A/G	0.13	3′-UTR affecting RNA expression; associated with an increased risk of IPA infection	[12]
DC-SIGN_c.342+2863	rs8112310	5′ near gene	–	A/T	0.16	Potential activity affecting DC-SIGN expression	–
DC-SIGN_IVS6 -326	rs10410342	Intron	–	C/G	0.07	Unknown	–
DC-SIGN_ c.749-28	rs11465384	Intron	–	C/T	0.09	Associated with an increased risk of IPA infection	[12]
DC-SIGN_ c.1974	rs11465413	3′-UTR	–	A/T	0.10	3′-UTR affecting RNA expression	–
DC-SIGN_IVS2+11	rs7252229	Intron	–	G/C	0.16	Associated with an increased risk of IPA infection	[12]
DC-SIGN_c.898	rs7248637	3′-UTR	–	A/G	0.11	3′-UTR affecting RNA expression; associated with an increased risk of IPA infection	[12]
DC-SIGN_c.2629	rs11465421	3′-UTR	–	A/C	0.42	3′-UTR affecting RNA expression	–
Dectin-1 (CLEC7A)_c.714	rs16910526	Coding exon	Y238X	A/C	0.08	Defective expression of Dectin-1, lack of b-glucan recognition by phagocytes and defectiveproduction of cytokines; associated with increased Aspergillus and Candida colonization inhematopoietic transplant recipients	[13]–[15]
Dectin-1 (CLEC7A)_c.375-1148	rs11053599	Intron	–	A/C		Unknown	–
Dectin-1 (CLEC7A)_c.375-1404	rs7309123	Intron	–	C/G	0.42	Associated with an increased risk of IPA infection	[12]
Dectin-1 (CLEC7A)_c.255+813	rs3901533	Intron	–	G/T	0.24	Associated with an increased risk of IPA infection	[12]
Dectin-1 (CLEC7A)_c.104-520	rs4763446	Intron	–	C/T	0.15	Unknown	–
Dectin-1 (CLEC7A)_c.104-811	rs16910631	Intron	–	C/T	0.07	Unknown	–
Dectin-1 (CLEC7A)_c.103+732	rs7311598	Intron	–	A/G	0.16	Unknown	–
Dectin-2 (CLEC6A)_c.369+338	rs7134303	Intron	–	A/G	0.18	Unknown	–
Dectin-2 (CLEC6A)_c.122-425	rs4264222	Intron	–	C/T	0.22	Unknown	–
Dectin-2 (CLEC6A)_c.32-699	rs4459385	Intron	–	C/T	0.25	Unknown	–
MCP-1 (CCL2)_c.903	rs4586	Coding exon	C35C	C/T	0.48	Associated with an increased risk of TB	[16], [17]
MCP-1 (CCL2)_c.-2136	rs1024610	Promoter	–	A/T	0.23	Unknown	–
MCP-1 (CCL2)_c.-2518	rs1024611	Promoter	–	C/T	0.25	Correlate with MCP-1 mRNA expression and influence on the risk of TB,asthma, COPD, HCV and HBV infections	[16], [18]–[23]
MCP-1 (CCL2)_c.1543	rs13900	3′-UTR	–	C/T	0.24	Unknown	–
CCR2 _c.-1221	rs3918358	Promoter	–	A/C	0.31	Unknown	–
CCR2 _c.667	rs743660	3′-UTR	–	A/G	0.25	Associated with an increased risk for COPD	
CCR2_Ex2+241	rs1799864	Coding exon	V64I	A/G	0.10	Associated with slower progression to HIV	[23], [24]

Abbreviations: UTR, untranslated region; IPA, Invasive Pulmonary Aspergillosis; TB, Tuberculosis; COPD, Chronic obstructive pulmonary disease; HCV, Hepatitis C virus; HBV, Hepatitis B virus; HIV-1, Human immunodeficiency virus-1; SARS, acute severe respiratory syndrome.

‡ Minor allele frequency found in our population (458 RA patients and 512 controls).

* Tagging details and references are included as Supplementary material.

### Statistical Analysis

The Hardy-Weinberg Equilibrium (HWE) tests were performed in the control group by a standard observed-expected chi-square (χ^2^) test at each polymorphic site (http://ihg2.helmholtz-muenchen.de/cgi-bin/hw/hwa1.pl). Binary logistic regression was used to assess the main effects of the genetic polymorphisms on RA risk using co-dominant and dominant inheritance models. For each SNP, the more common allele in the control group was assigned as the reference category. Binary logistic regression analyses were adjusted for age and gender whereas gender-stratified analyses were adjusted for age. In the pooled analysis, overall binary logistic regression analyses were adjusted by age, gender and centre whereas gender-stratified analyses were adjusted for age and centre. Statistical power was estimated using Quanto software (http://hydra.usc.edu/gxe/). All analyses were conducted using the statistical software SSPS (version 14.0, SPSS Inc., Chicago, USA). All tests were considered to be statistically significant when p<0.05. In order to correct for multiple testing, we calculated an adjusted significance level using M_eff_ correction. We estimated an individual M_eff_ value for each gene (i.e. the effective number of independent variables) and the study-wise M_eff_ value was estimated by adding up the independent gene M_eff_ values by using the SNP Spectral Decomposition Lite program (http://gump.qimr.edu.au/general/daleN/SNPSpDsuperlite/) [27]. The resulting number of independent marker loci was applied to correct for multiple testing.

### Functional Prediction of Associated SNPs

We used a web-based tool FastSNP (available at http://fastsnp.ibms.sinica.edu.tw) for predicting the functional significance of the SNPs associated with RA. A detailed explanation on the FastSNP tool has been described elsewhere [28]. FastSNP utilizes information from different web resources (Hapmap, dbSNP, NCBI Blast, Polyphen, SNPs3D, TRANSFAC, ESEfinder, Rescue-ESE and FAS-ESS) to determine whether SNPs are located at exonic splicing regulatory sites or whether they alter the transcription factor-binding site of a gene (for instance, acting as intronic enhancer) or even whether SNPs are affecting Micro-RNA binding sites. In addition, these tools are able to identify either nonsense or non-synonymous SNPs that lead to premature termination of translation or amino acid changes and that are, therefore, very likely to affect protein function.

### SNP-SNP Interaction Analysis

We also analyzed high-order interactions between significant SNPs using the multifactor dimensionality reduction (MDR) constructive induction algorithm. A detailed explanation on the MDR method has been described elsewhere [29]. Cross-validation and permutation testing were used to identify the best models. All possible two-way SNP interactions were tested using 10-fold cross-validation and the exhaustive search. The model with the highest testing balanced accuracy (TA) and cross validation consistency (CVC) was selected as “best model”. Statistical significance was evaluated by the Sign test and confirmed using a 1.000-fold permutation test to compare observed testing balanced accuracies with those expected under the null hypothesis of no association (using the MDR permutation testing module 0.4.9 alpha) [29]. MDR results were considered statistically significant at the level of 0.05. MDR software and MDR permutation testing module are open-source and freely available from http://www.epistasis.org. Logistic regression analyses were also performed to confirm significant interaction results from MDR analyses.

## Results

Demographic and clinical characteristics of the RA patients analysed in phase 1 are described in Table 2. Controls were slightly younger than RA patients (53.46±9.69 vs. 58.51±13.13; p<0.001). Seventy-eight percent of RA patients were female, 70.7% were positive for anti-CCP antibodies and 71.2% were positive for RF (rheumatoid factor). According to the DAS28, male patients showed low active disease (DAS28_Male_ = 2.89) whereas women had moderately active disease (DAS28_Female_ = 3.42). Women tended to have more often a DAS28≥3.2 compared to men (56.18% vs. 43.48%, p = 0.078). The overall average disease duration was 12.39 years.

**Table 2:** Demographic and clinical characteristics of RA patients.

	*RA patients*		
	*Overall (n = 458)*	*Women (n = 360)*	*Men (n = 98)*
*Demographic characteristics*			
* Age (years)*	58.51±13.13	57.94±13.02	60.56±13.39
*Clinical assessment*			
* Disease duration (years)*	12.39±7.62	12.64±7.84	11.43±6.61
* Percentage of patients with RF positivity*	71.20	70.26	74.72
* Percentage of patients with positive anti-CCP* *	70.70	71.65	66.67
* Current DAS28 (average)*	3.31	3.42	2.89
*Treatments*			
*DMARDs*			
* Methotrexate (%)*	259 (56.55)	207 (57.50)	52 (53.06)
* Leflunomide (%)*	178 (38.86)	132 (36.67)	46 (46.94)
* Sulphasalazine (%)*	40 (8.73)	31 (8.61)	9 (9.18)
*Biologic agents*			
* Infliximab (%)*	171 (37.34)	141 (39.17)	30 (30.61)
* Etanercept (%)*	140 (30.57)	116 (32.22)	24 (24.49)
* Adalimumab (%)*	124 (27.07)	102 (28.33)	22 (22.45)
* Abatacept (%)*	43 (9.39)	35 (9.72)	8 (8.16)
* Rituximab (%)*	112 (24.45)	91 (25.28)	21 (21.42)
* Tocilimumab (%)*	20 (4.37)	18 (5.00)	2 (2.04)
* Others (%)*	7 (1.52)	6 (1.67)	1 (1.02)
*Number of biologic agents*			
* 0*	114 (24.89)	76 (21.11)	38 (38.77)
* 1*	195 (42.58)	162 (45.00)	33 (33.67)
* 2*	71 (15.50)	56 (15.55)	15 (15.31)
* 3*	46 (10.04)	40 (11.11)	6 (6.12)
* 4*	20 (4.37)	17 (4.72)	3 (3.06)
* >4*	12 (2.62)	9 (2.50)	3 (3.06)

Data are means ± standard deviation. Abbreviations: RF, rheumatoid factor; Anti-CCP: anti-cyclic citrullinated peptide antibodies; DAS28, disease activity score; DMARDs, disease-modifying antirheumatic drugs.

* Anti-CCP value was available only in 314 patients (254 women and 60 men).

All analyzed SNPs were in HWE in the control group with the exception of *Dectin-1*_rs16910631_ (p>0.01). Five SNPs showed overall association with RA (Table 3 and Table S1). Carriers of *Dectin-2*_rs4264222T_ allele had an increased risk of RA (OR = 1.47, 95%CI 1.10–1.96, *P* = 0.009) whereas patients harboring the *DC-SIGN*_rs4804803G_, *MCP-1*_rs1024611G_, *MCP-1*_rs13900T_ and *MCP-1*_rs4586C_ alleles had a decreased risk of developing the disease (OR = 0.66, 95%CI 0.49–0.88, *P* = 0.004; OR = 0.66, 95%CI 0.50–0.89, P = 0.006; OR = 0.73, 95%CI 0.55–0.97, *P* = 0.03 and OR = 0.68, 95%CI 0.51–0.91, P = 0.009; Table 3). In addition, patients bearing the *Dectin-2*_rs7134303G_ allele showed a trend to have an increased risk for RA (OR = 1.35, 95%CI 1.00–1.83). Most importantly, binary logistic regression analyses revealed gender-specific associations with RA for six SNPs (Table 3). There was a significant effect modification by gender for *Dectin-2*_rs4264222_ and *Dectin-2*_rs7134303_ SNPs (*P*_interaction_ = 0.041 and 0.011, respectively; Table 3). Women carrying either the *Dectin-2*_rs4264222T_ or *Dectin-2*_rs7134303G_ alleles had an increased risk of RA (OR = 1.93, 95%CI 1.34–2.79 and OR = 1.90, 95%CI 1.29–2.80). Additionally, women carrying the *MCP-1*_rs1024611G_, *MCP-1*_rs13900T_ and *MCP-1*_rs4586C_ alleles had a decreased risk of RA compared with those carrying the wild-type genotype (OR = 0.61, 95%CI 0.43–0.87; OR = 0.67, 95%CI 0.47–0.95 and OR = 0.60, 95%CI 0.42–0.86). As also men had decreased, but statistically not significant ORs, no effect modification by gender was observed. A borderline gender-specific effect was observed for the *DC-SIGN*_rs2287886_ SNP (*P*_interaction_ = 0.071; Table 3). Here, male carriers of the *DC-SIGN*_rs2287886A_ allele had an increased risk of RA (OR = 1.70, 95%CI 1.03–2.78). Additionally, the association of the *DC-SIGN*_rs4804803G_ with a decreased risk was stronger in men than in women (OR = 0.53, 95%CI 0.32–0.89 vs. OR = 0.73, 95%CI 0.51–1.04).

**Table 3:** Dectin-2, DC-SIGN and MCP-1 polymorphisms associated with rheumatoid arthritis.

	*Overall (n = 970)*				*Men (n = 393)*				*Women (n = 577)*				
Variant information	Control (%)	Cases (%)	OR (95% CI)^1^	*P value*	Control (%)	Cases (%)	OR (95% CI)^2^	*P value*	Control (%)	Cases (%)	OR (95% CI)^2^	*P value*	*P* *interaction* 3
*DC-SIGN_*rs2287886													
G/G	242 (49.0)	199 (44.8)	1.00		141 (49.8)	38 (40.0)	1.00		101 (47.9)	161 (46.1)	1.00		
A/G	201 (40.7)	191 (43.0)	1.26 (0.93–1.70)		119 (42.0)	42 (44.2)	1.52 (0.90–2.57)		82 (38.9)	149 (42.7)	1.16 (0.80–1.69)		
A/A	51 (10.3)	54 (12.2)	1.20 (0.75–1.91)	0.32	23 (8.1)	15 (15.8)	**2.56 (1.16–5.63)**	**0.05**	28 (13.3)	39 (11.2)	0.83 (0.48–1.45)	0.47	0.071
G/G vs. A/G+A/A	252 (51.0)	245 (55.2)	1.24 (0.94–1.65)	0.13	142 (50.2)	57 (60.0)	**1.70 (1.03–2.78)**	**0.04**	110 (52.1)	188 (53.9)	1.08 (0.76–1.53)	0.67	
*DC-SIGN_*rs4804803													
A/A	270 (54.2)	285 (63.9)	1.00		153 (53.7)	66 (68.8)	1.00		117 (54.9)	219 (62.6)	1.00		
A/G	193 (38.8)	135 (30.3)	**0.64 (0.47–0.86)**		108 (37.9)	27 (28.1)	0.59 (0.34–1.01)		85 (39.9)	108 (30.9)	**0.67 (0.46–0.97)**		
G/G	35 (7.0)	26 (5.8)	0.79 (0.44–1.42)	**0.01**	24 (8.4)	3 (3.1)	0.29 (0.08–1.04)	**0.03**	11 (5.2)	23 (6.6)	1.23 (0.57–2.65)	0.07	0.149
A/A vs. A/G+G/G	228 (45.8)	161 (36.1)	**0.66 (0.49–0.88)**	**0.004**	132 (46.3)	30 (31.2)	**0.53 (0.32–0.89)**	**0.02**	96 (45.1)	131 (37.4)	0.73 (0.51–1.04)	0.08	
*Dectin-2*_rs7134303													
A/A	350 (69.9)	287 (64.9)	1.00		188 (65.7)	70 (72.9)	1.00		162 (75.3)	217 (62.7)	1.00		
A/G	136 (27.1)	138 (31.2)	1.34 (0.98–1.84)		88 (30.8)	23 (24.0)	0.72 (0.41–1.26)		48 (22.3)	115 (33.2)	**1.88 (1.26–2.81)**		
G/G	15 (3.0)	17 (3.8)	1.41 (0.65–3.09)	0.15	10 (3.5)	3 (3.1)	0.71 (0.18–2.83)	0.48	5 (2.3)	14 (4.0)	2.08 (0.72–5.97)	**0.004**	**0.011**
A/A vs. A/G+G/G	151 (30.1)	155 (35.1)	1.35 (1.00–1.83)	**0.05**	98 (34.3)	26 (27.1)	0.72 (0.42–1.23)	0.22	53 (24.6)	129 (37.3)	**1.90 (1.29–2.80)**	**9.00E-04**	
*Dectin-2*_rs4264222													
C/C	324 (64.3)	254 (57.0)	1.00		173 (59.9)	60 (63.2)	1.00		151 (70.2)	194 (55.3)	1.00		
C/T	160 (31.8)	165 (37.0)	**1.44 (1.06–1.95)**		104 (36.0)	31 (32.6)	0.89 (0.53–1.50)		56 (26.1)	134 (38.2)	**1.90 (1.29–2.78)**		
T/T	20 (4.0)	27 (6.0)	1.67 (0.86–3.24)	**0.03**	12 (4.2)	4 (4.2)	1.03 (0.31–3.45)	0.91	8 (3.7)	23 (6.5)	2.19 (0.94–5.10)	**0.001**	**0.041**
C/C vs. C/T+T/T	180 (35.7)	192 (43.0)	**1.47 (1.10–1.96)**	**0.009**	116 (40.1)	35 (36.8)	0.91 (0.55–1.49)	0.70	64 (29.8)	157 (44.7)	**1.93 (1.34–2.79)**	**3.00E-04**	
*MCP-1*_rs1024611													
A/A	260 (54.5)	275 (61.8)	1.00		160 (58.4)	60 (64.5)	1.00		100 (49.3)	215 (61.1)	1.00		
G/A	178 (37.3)	139 (31.2)	**0.64 (0.47–0.88)**		90 (32.9)	24 (25.8)	0.80 (0.46–1.42)		88 (43.4)	115 (32.7)	**0.60 (0.41–0.87)**		
G/G	39 (8.2)	31 (7.0)	0.76 (0.44–1.31)	**0.02**	24 (8.8)	9 (9.7)	0.82 (0.35–1.93)	0.71	15 (7.4)	22 (6.2)	0.68 (0.34–1.39)	**0.02**	0.692
A/A vs. G/A+G/G	217 (45.5)	170 (38.2)	**0.66 (0.50–0.89)**	**0.006**	114 (41.6)	33 (35.5)	0.81 (0.48–1.35)	0.41	103 (50.7)	137 (38.9)	**0.61 (0.43–0.87)**	**0.006**	
*MCP-1*_rs13900													
C/C	271 (54.6)	276 (60.9)	1.00		164 (56.9)	60 (61.2)	1.00		107 (51.4)	216 (60.9)	1.00		
C/T	190 (38.3)	151 (33.3)	**0.71 (0.53–0.96)**		101 (35.1)	29 (29.6)	0.87 (0.51–1.49)		89 (42.8)	122 (34.4)	**0.66 (0.46–0.95)**		
T/T	35 (7.1)	26 (5.7)	0.84 (0.47–1.49)	0.08	23 (8.0)	9 (9.2)	0.90 (0.38–2.12)	0.88	12 (5.8)	17 (4.8)	0.74 (0.34–1.63)	0.08	0.704
C/C vs. C/T+T/T	225 (45.4)	177 (39.1)	**0.73 (0.55–0.97)**	**0.03**	124 (43.1)	38 (38.8)	0.88 (0.54–1.44)	0.61	101 (48.6)	139 (39.1)	**0.67 (0.47–0.95)**	**0.03**	
*MCP-1*_rs4586													
T/T	191 (39.5)	209 (46.4)	1.00		121 (43.8)	47 (48.0)	1.00		70 (33.6)	162 (46.0)	1.00		
C/T	227 (46.9)	190 (42.2)	**0.68 (0.50–0.92)**		117 (42.4)	37 (37.8)	0.82 (0.49–1.39)		110 (52.9)	153 (43.5)	**0.61 (0.42–0.89)**		
C/C	66 (13.6)	51 (11.3)	0.69 (0.44–1.09)	**0.03**	38 (13.8)	14 (14.3)	0.98 (0.47–2.05)	0.75	28 (13.5)	37 (10.5)	**0.56 (0.31–0.99)**	**0.02**	0.409
T/T vs. C/T+C/C	293 (60.5)	241 (53.6)	**0.68 (0.51–0.91)**	**0.009**	155 (56.2)	51 (52.0)	0.86 (0.53–1.40)	0.55	138 (66.3)	190 (54.0)	**0.60 (0.42–0.86)**	**0.005**	

1 Models adjusted for age and gender.

2 Models adjusted for age.

3 *p* value for testing of effect modification by gender was calculated utilizing an interaction term of gender and genetic polymorphism assuming a co-dominant model of inheritance. *P*<0.05 in bold. Abbreviations: OR, odds ratio; CI, confidence interval.

After correction for multiple testing using the SNP Spectral Decomposition Lite program (number of independent marker loci, 21; p = 0.05/21 = 0.002), only the *Dectin-2*_rs4264222_ and *Dectin-2*_rs7134303_ associations in women retained significance (*P* = 3.00 E-04 and *P* = 9.00 E-04), whereas the associations with *DC-SIGN*_rs4804803,_ *MCP-1*_rs1024611_ and *MCP-1*_rs4586_ showed borderline significance in the whole population (*P* = 0.004, *P* = 0.006 and *P* = 0.009; Table 3).

In phase 2, in order to increase the statistical power of this study and to confirm the significant associations, we further genotyped the significant SNPs in 754 Caucasian RA patients (626 women and 128 men) and 519 healthy controls (348 women and 171 men). Clinical characteristics of the RA patient population are shown in Table S2 and characteristics of the pooled population are shown in Table S3. In the phase 2, RA patients had a similar age and showed slightly higher percentages of anti-CCP (77.72 vs. 70.70) and RF positivity (75.98 vs. 71.20). As before, male patients showed low active disease when compared to women (DAS28_Male_ = 3.21 vs. DAS28_Female_ = 3.78). Thus, we end up with a total population of 2.243 individuals including 1.212 RA patients (986 women and 226 men) and 1.031 controls (565 women and 466 men). After this recruitment the study had over 80% power (co-dominant model) to detect an odds ratio of 1.19 at alpha = 0.008 (multiple testing threshold) for a polymorphism with a minor allele frequency of 0.25. Although the gender-stratified analysis reduced the statistical power of the study, we still had power to detect reasonably small risks (OR_WOMEN_ = 1.23 and OR_MEN_ = 1.36).

Binary logistic regression analysis adjusted for age, gender and center in the pooled population showed that *MCP-1*_rs1024611_, *MCP-1*_rs13900_, *MCP-1*_rs4586_ and *DC-SIGN*_rs4804803_ polymorphisms were associated with a decreased risk of developing RA (OR = 0.76, 95%CI 0.61–0.95, *P* = 0.01; OR = 0.77, 95%CI 0.62–0.96, *P* = 0.02; OR = 0.80, 95%CI 0.64–0.99, *P* = 0.04; and OR = 0.77, 95%CI 0.62–0.96, *P* = 0.02; Table 4). Although there was no significant effect modification by gender, *Dectin-2* and *MCP-1* polymorphisms seemed to have an stronger effect in women compared to men, whereas variants within *DC-SIGN* showed more evident effects in men. Thus, women carrying the *Dectin-2*_rs4264222T_ allele had an increased risk of RA (OR = 1.38, 95%CI 1.08–1.77, *P* = 0.01) whereas women harbouring the *MCP-1*_rs1024611G_ and *MCP-1*_rs13900T_ alleles had a decreased risk of RA in comparison with those carrying the wild-type genotype (OR = 0.74, 95%CI 0.58–0.94, *P* = 0.02 and OR = 0.76, 95%CI 0.59–0.97, *P* = 0.03; Table 4). Women bearing the *Dectin-2*_rs7134303G_ showed also a trend towards an increased risk of the disease (OR = 1.30, 95%CI 1.00–1.69, *P* = 0.05) whereas those women carrying the *MCP-1*_rs4586C_ allele had a decreased risk for RA (OR = 0.78, 95%CI 0.61–1.00, *P* = 0.05; Table 4). We could confirm that none of these effects was observed in men. We also confirmed that men carrying the *DC-SIGN*_rs4804803G_ allele had a decreased risk to develop RA (OR = 0.56, 95%CI 0.34–0.93, *P* = 0.02; Table 4) whereas men harboring *DC-SIGN*_rs2287886A_ allele had trend to have an increased risk of RA (OR = 1.54, 95%CI 0.96–2.46, *P* = 0.07). None of these associations resisted multiple testing adjustments in the pooled sample (number of independent marker loci was estimated including only those SNPs genotyped in the pooled sample; p = 0.05/6 = 0.008) and all require further replication in independent populations.

**Table 4:** Overall analysis of Dectin-2, DC-SIGN and MCP-1 polymorphisms with rheumatoid arthritis.

	*Overall (n = 2252)*				*Men (n = 692)*				*Women (n = 1560)*				
Variant information	Control (%)	Cases (%)	OR (95% CI)^1^	*P value*	Control (%)	Cases (%)	OR (95% CI)^2^	*P value*	Control (%)	Cases (%)	OR (95% CI)^2^	*P value*	*P interaction* 3
*DC-SIGN_*rs2287886													
G/G	460 (47.5)	534 (46.1)	1.00		209 (48.6)	90 (41.7)	1.00		251 (46.6)	444 (47.1)	1.00		
A/G	389 (40.2)	487 (42.1)	1.22 (0.97–1.53)		178 (41.4)	96 (44.4)	1.42 (0.86–2.35)		211 (39.2)	391 (41.5)	1.16 (0.89–1.51)		
A/A	119 (12.3)	137 (11.8)	1.10 (0.78–1.53)	0.25	43 (10.0)	30 (13.9)	1.98 (0.95–4.12)	0.14	76 (14.1)	107 (11.4)	0.91 (0.63–1.33)	0.36	0.149
G/G vs. A/G+A/A	508 (52.5)	624 (53.9)	1.19 (0.96–1.47)	0.12	221 (51.4)	126 (58.3)	1.54 (0.96–2.46)	0.07	287 (53.4)	497 (52.8)	1.09 (0.86–1.39)	0.47	
*DC*–*SIGN_*rs4804803													
A/A	557 (57.1)	730 (62)	1.00		244 (56.2)	142 (64.8)	1.00		313 (57.9)	588 (61.3)	1.00		
A/G	350 (35.9)	370 (31.4)	**0.74 (0.59–0.93)**		155 (35.7)	66 (30.1)	**0.58 (0.34–0.99)**		195 (36)	304 (31.7)	0.77 (0.59–1.00)		
G/G	68 (7)	78 (6.6)	0.93 (0.60–1.43)	**0.04**	35 (8.1)	11 (5)	0.48 (0.18–1.30)	0.07	33 (6.1)	67 (7)	1.09 (0.65–1.81)	0.11	0.413
A/A vs. A/G+G/G	418 (42.9)	448 (38)	**0.77 (0.62–0.96)**	**0.02**	190 (43.8)	77 (35.2)	**0.56 (0.34–0.93)**	**0.02**	228 (42.1)	371 (38.7)	0.81 (0.64–1.04)	0.10	
*Dectin-2*_rs7134303													
A/A	645 (68)	715 (62.3)	1.00		285 (66.3)	134 (64.1)	1.00		360 (69.4)	581 (61.9)	1.00		
A/G	269 (28.4)	385 (33.6)	1.15 (0.91–1.45)		126 (29.3)	67 (32.1)	0.68 (0.39–1.17)		143 (27.6)	318 (33.9)	**1.32 (1.01–1.73)**		
G/G	35 (3.7)	47 (4.1)	0.87 (0.49–1.54)	0.42	19 (4.4)	8 (3.8)	0.31 (0.08–1.29)	0.11	16 (3.1)	39 (4.2)	1.15 (0.58–2.26)	0.13	0.096
A/A vs. A/G+G/G	304 (32)	432 (37.7)	1.11 (0.89–1.40)	0.36	145 (33.7)	75 (35.9)	0.62 (0.37–1.06)	0.08	159 (30.6)	357 (38.1)	1.30 (1.00–1.69)	**0.05**	
*Dectin-2*_rs4264222													
C/C	623 (62.2)	647 (56.3)	1.00		272 (59.9)	121 (56.8)	1.00		351 (64.0)	526 (56.1)	1.00		
C/T	326 (32.5)	434 (37.7)	1.26 (1.00–1.58)		157 (34.6)	81 (38)	0.86 (0.52–1.42)		169 (30.8)	353 (37.7)	**1.40 (1.08–1.82)**		
T/T	53 (5.3)	69 (6.0)	1.11 (0.69–1.77)	0.14	25 (5.5)	11 (5.2)	0.79 (0.26–2.36)	0.80	28 (5.1)	58 (6.2)	1.26 (0.74–2.16)	**0.04**	0.219
C/C vs. C/T+T/T	379 (37.8)	503 (43.7)	1.24 (1.00–1.53)	0.06	182 (40.1)	92 (43.2)	0.85 (0.52–1.38)	0.51	197 (36.0)	411 (43.9)	**1.38 (1.08–1.77)**	**0.01**	
*MCP-1*_rs1024611													
A/A	522 (55.3)	672 (57.9)	1.00		245 (58.8)	126 (58.3)	1.00		277 (52.6)	546 (57.8)	1.00		
G/A	352 (37.3)	406 (35.0)	**0.73 (0.58–0.92)**		135 (32.4)	71 (32.9)	0.78 (0.45–1.36)		217 (41.2)	335 (35.5)	**0.71 (0.55–0.92)**		
G/G	70 (7.4)	82 (7.1)	0.94 (0.61–1.43)	**0.03**	37 (8.9)	19 (8.8)	0.89 (0.39–2.01)	0.68	33 (6.3)	63 (6.7)	0.94 (0.56–1.56)	**0.03**	0.604
A/A vs. G/A+G/G	422 (44.7)	488 (42.1)	**0.76 (0.61–0.95)**	**0.01**	172 (41.2)	90 (41.7)	0.81 (0.49–1.33)	0.40	250 (47.4)	398 (42.2)	**0.74 (0.58–0.94)**	**0.02**	
*MCP-1*_rs13900													
C/C	547 (55.4)	679 (57.7)	1.00		262 (58.5)	129 (58.6)	1.00		285 (52.9)	550 (57.5)	1.00		
C/T	379 (38.4)	416 (35.4)	**0.74 (0.59–0.93)**		153 (34.1)	73 (33.2)	0.81 (0.48–1.37)		226 (41.9)	343 (35.9)	**0.73 (0.56–0.93)**		
T/T	61 (6.2)	81 (6.9)	0.99 (0.64–1.54)	**0.03**	33 (7.4)	18 (8.2)	0.86 (0.37–2.03)	0.72	28 (5.2)	63 (6.6)	1.02 (0.60–1.74)	**0.04**	0.774
C/C vs. C/T+T/T	440 (44.6)	497 (42.3)	**0.77 (0.62–0.96)**	**0.02**	186 (41.5)	91 (41.4)	0.82 (0.51–1.33)	0.43	254 (47.1)	406 (42.5)	**0.76 (0.59–0.97)**	**0.03**	
*MCP-1*_rs4586													
T/T	396 (40.9)	498 (42.1)	1.00		195 (44.8)	94 (42.5)	1.00		201 (37.7)	404 (42.0)	1.00		
C/T	455 (47.0)	526 (44.5)	**0.74 (0.59–0.94)**		181 (41.6)	93 (42.1)	0.64 (0.38–1.09)		274 (51.4)	433 (45.1)	**0.74 (0.57–0.96)**		
C/C	117 (12.1)	158 (13.4)	1.03 (0.73–1.45)	**0.02**	59 (13.6)	34 (15.4)	1.22 (0.61–2.43)	0.13	58 (10.9)	124 (12.9)	0.95 (0.63–1.43)	0.07	0.793
T/T vs. C/T+C/C	572 (59.1)	684 (57.9)	**0.80 (0.64–0.99)**	**0.04**	240 (55.2)	127 (57.5)	0.77 (0.48–1.24)	0.28	332 (62.3)	557 (58.0)	0.78 (0.61–1.00)	**0.05**	

1 Models adjusted for age, gender and center.

2 Models adjusted for age and center.

3 *p* value for testing of effect modification by gender was calculated utilizing an interaction term of gender and genetic polymorphism assuming a co-dominant model of inheritance. *P*<0.05 in bold. Abbreviations: OR, odds ratio; CI, confidence interval.

For predicting the effect of polymorphisms found associated with RA we used FastSNP, which predicts the possible effect of genetic variants on the protein function and/or structure. Among the SNPs associated either with increased or decreased risk of RA, 2 SNPs were predicted to have functional impact. The predictive functional analysis suggested an intronic enhancer function for *Dectin-2*_rs7134303_ due to its location in a transcription factor-binding site (risk score 1–2) and a function as gene expression regulator for the *MCP-1*_rs1024611_ (risk score 1–3). The presence of the *Dectin-2*_rs7134303G_ allele creates a nuclear protein-binding site for the transcription factor GATA-1 (GATA-binding factor 1) whereas the presence of the *MCP-1*_rs1024611T_ allele, which confers protection against RA, disrupts a binding site for both GATA-1 and GATA-2 in the promoter region of the MCP-1 gene. Whereas GATA-1 is a transcription factor expressed in circulating inflammatory monocytes and indispensable for effective DC maturation and survival, GATA-2 expression inhibits monocyte differentiation. These data suggest a central role of the *Dectin-2*_rs7134303_ and *MCP-1*_rs1024611_ polymorphisms in the gender-associated susceptibility to RA and point out a possible participation of GATA-1 and GATA-2 in the modulation of *Dectin-2* and *MCP-1* expression and, consequently, in the activation of both monocytes and DCs.

We also investigated the epistatic effect of the significant SNPs in order to find possible gender-specific high order interactions. A summary of the results for the models that had maximum testing accuracy and maximum cross validation consistency in women and men is presented in Table 5. In women, the overall best model consisted of *Dectin-2*_rs4264222_ and *Dectin-2*_rs7134303_ SNPs that interacted in a synergistic or non-additive manner. Logistic regression analysis applied to this two -locus model confirmed our results (*P* = 0.007). In men, no SNP-SNP interactions were shown to be significant (Table 5).

**Table 5:** MDR analysis to detect two-locus disease models.

*Women*		*Gene*	*Model*	*TA*	*Sing test (P-value)*	*P-value* *	*CVC*
	1	*Dectin-2*	rs7134303	0.5402	3 (*P* = 0.95)	NS	9/10
	2	*Dectin-2*	rs4264222, rs7134303	0.5560	8 (*P* = 0.05)	0.007	9/10
***Men***		***Gene***	***Model***	***TA***	***Sing test (P-value)***	***P-value*** *	***CVC***
	1	*DC-SIGN*	rs2287886	0.5424	9 (*P* = 0.01)	NS	10/10
	2	*MCP-1, DC-SIGN*	rs13900, rs2287886	0.4738	5 (*P* = 0.62)	NS	3/10

TA, Testing accuracy; CVC, Cross-validation consistency. P<0.05 was considered significant. NS, not significant.

* P-value for testing balanced accuracy using 1.000-fold permutation test (MDR permutation testing module vs.0.4.9 alpha).

Finally, we explored the effect of selected SNPs on disease activity and severity. No differences were found in relation to DAS28, the positivity for anti-CCP antibodies or RF when RA patients were grouped by gender and genotype (data not shown).

## Discussion

The idea that genetic factors have an impact on the predisposition to RA is well supported by our understanding of the disease biology and by data from a wide range of genetic epidemiologic studies [30]–[32]. This fact, along with studies showing sex-specific differences in incidence of RA and in immune response [10], [33], suggests that genetic factors modulating the immune response may contribute to the sex-specific incidence rates. Considering this hypothesis, the present study was carried out to assess whether genetic variants within immune-related genes may differentially play a role in determining the risk of RA in women and men. Although to date there have been too few studies addressing the effect of gender on the risk of RA, some previous studies have reported female-specific associations between genetic variants in immune genes (TNF, TNFR2, IL4R and CD4) and risk of RA [23], [34], [35].

The present population-based case-control study included 2.243 individuals (1.212 RA patients and 1.031 controls) and confirmed the role of 4 SNPs within *Dectin-2*, *MCP-1* and *DC-SIGN* genes in determining the risk of RA. These results were in agreement with a previous study performed by Platinga *et al.* (2010), who reported no overall association of *Dectin-1*_rs16910526_ SNP with RA [36] but were in contrast with those results described by Dieguez-González *et al.* (2009) reporting no association of the *DC-SIGN*_rs4804803_ and RA [37]. These controversial results might be attributed to differences in population size and environmental factors but also confounding factors such as gender.

We also identified, for the first time, genetic variants in *Dectin-2, DC-SIGN* and *MCP-1* as contributing to the gender-specific risk for RA. Females with the *Dectin-2*_rs4264222T_ allele had an increased risk to develop RA whereas females carrying any of the *MCP-1*_rs1024611G_ and *MCP-1*_rs13900T_ alleles had a decreased risk to develop RA compared with carriers of the wild-type genotype. We also found that women bearing the *Dectin-2*_rs7134303G_ tended to have an increased risk of the disease whereas those women carrying the *MCP-1*_rs4586C_ allele showed a borderline significant decreased risk for RA. In males, none of these effects were found but carriers of the *DC-SIGN*_rs4804803G_ had a significantly decreased risk of developing the disease whereas those harboring the *DC-SIGN*_rs2287886A_ allele had trend to have an increased risk of RA. Although there was not significant effect modification by gender, *Dectin-2*, *MCP-1* and *DC-SIGN* polymorphisms showed consistent associations in women that were not seen in men and vice versa. Of note is that, after adjusting the significance level to account for multiple comparisons (study-wise significant *P*-threshold = 0.008), *Dectin-2*_rs4264222_, *MCP-1*_rs1024611_, *MCP-1*_rs13900_ and *MCP-1*_rs4586_ SNPs showed both overall and gender-specific borderline significant associations. These results underlie the potential importance of analyzing RA data both with and without gender as a stratifying factor.

Gender-specific SNP-SNP interactions among the selected variants indicated that the combined effect of the polymorphisms with or without significant main effects conferred risk for RA. The MDR approach used in this study identified a two-locus significant model associated with high risk of RA in women while no significant models were found in men. In women, the strongest model for predicting RA risk was a model including *Dectin-2*_rs4264222_ and *Dectin-2*_rs7134303_ SNPs that interact in synergistic manner to increase the risk of RA. Although interesting, these results should be interpreted with caution given that the statistical modeling of SNP-SNP interactions may not be assumed as a true biological interaction. However, based on *in silico* tools, hypotheses concerning the molecular mechanisms resulting in differential activity of genes can be created. In this regard, these two SNPs (*Dectin-2*_rs7134303_ and *Dectin-2*_rs4264222_) were found to be important in this study, both in the single SNP or the epistatic analysis. MDR analysis was further confirmed by logistic regression analysis. *Dectin-2*_rs7134303_ creates an allele-specific change in transcriptional binding site for GATA-1 and could contribute, acting as intronic enhancer, to the transcriptional regulation of *Dectin-2* gene and, consequently, to affect its intracellular signaling and downstream gene expression.

According to these results, it is conceivable to suggest that *Dectin-2* variants, independently and/or through interactions may, at least partially, account for gender differences in susceptibility to RA. *Dectin-2*, a type II transmembrane receptor, is mainly expressed in DCs, macrophages and B-cells and its expression may be induced by inflammatory stimuli [38]. It is well known that *Dectin-2* recognizes carbohydrate motifs of self and non-self antigens. However, it has also been suggested that *Dectin-2* binds to endogenous ligands in CD4^+^CD25^+^ [39] and CD8^+^ T-cells [40] thus triggering inflammatory responses. Most recently, *Dectin-2* has been involved in promoting *Syk*- and *CARD9*-dependent *NFkB* activation [41], inducing the expression of a wide variety of pro-inflammatory mediators including cytokines, chemokines and co-stimulatory molecules and, thereby enhancing T-cell mediated immune responses. In line with this, Sato *et al.* also reported that, contrary to *Dectin-1* that binds *Syk* directly, *Dectin-2* couples to *Syk* through its association with Fc receptor gamma (*FcRγ*) chain leading to the activation of *NFkB* pathway and production of cytokines such as *TNF* and *IL1RA* [42]. Interestingly, it has also been described that *Dectin-2* is involved in the modulation of Th17 immune responses and that the production of *IL1β* and *IL23* upon *Dectin-2* requires *c-Rel*-dependent activation of *NFkB* [43]. These findings, along with those suggesting the role of CD4^+^ T cells secreting *IL17* (Th17 cells) [44] and *NFkB* activation in the pathogenesis and/or progression of RA [45], may indicate a key role for *Dectin-2* in the exacerbation of the immune response that define the RA. Nonetheless, the molecular mechanisms by which the *Dectin-2*_rs7134303_ and *Dectin-2*_rs4264222_ polymorphisms might increase the risk for developing RA remain to be clarified. Based on the findings reported by Gutierrez *et al.* (2007) that demonstrate the expression of GATA-1 in circulating inflammatory monocytes and its role in the maturation and survival of DCs [46] and given our *in silico* predictions that the *Dectin-2*_rs7134303_G_ allele might create a binding site for this transcription factor, we might hypothesize that this SNP could regulate *Dectin-2* expression and, consequently, to promote *Dectin-2*-induced *NFkB* activation and production of cytokines such as *TNF* and *IL1RA*, the key molecules in the ethiopathogenesis of RA. However, we cannot rule out the possibility that *Dectin-2*_rs7134303_ and *Dectin-2*_rs4264222_ polymorphisms may be in linkage disequilibrium with unidentified susceptible variants that are responsible for the significant associations observed.

*MCP-1/CCL2* is a modulator of monocyte/macrophage recruitment to the site of inflammation and subsequent T-cell activation [8] that acts through the CC chemokine receptor 2 (*CCR2*) [47]. *MCP-1* is involved in the promotion of leukocyte infiltration during the inflammation process and it has been found highly expressed in the synovial fluid of RA patients [7]. Several polymorphisms have been identified in the gene encoding *MCP-1* and some of them have previously been studied in relation to RA susceptibility [48],[49]. So far, most of these studies have been focused only on a potentially functional SNP, the *MCP-1*_rs1024611_ (-2518A/G) [24], [25], but no association with RA was found in Caucasian [48] or non-Caucasian populations [49], [50]. Conversely to these studies, we found an overall protective effect of the *MCP-1*_rs1024611_, *MCP-1*_rs13900_ and *MCP-1*_rs4586_ polymorphisms on the development of RA. However, given that the effect of these SNPs was restricted to women and given that these previous studies were based on a small population size, these studies might not have had sufficient statistical power to detect this association. Our predictive functional analysis also revealed that *MCP-1*_rs1024611_ disrupts the binding site for GATA-1 and GATA-2, which support our hypothesis suggesting a central role of these transcription factors mediating monocyte and DC maturation by controlling *MCP-1* and *Dectin-2* expression.

Similarly to other case-control studies, this study has several potential limitations. First, the statistical power was limited when a gender-stratified analysis was performed. To overcome this problem, we reported dominant rather than only co-dominant model. This limitation has also to be considered when analyzing SNP-SNP interactions as the combined analysis reduced some genotype groups to a few individuals. Finally, another limitation of this study is that no *in vivo* mechanisms were clarified in relation to *Dectin-2*, *DC-SIGN* and *MCP-1* variants.

## Conclusions

In conclusion, this study represents a preliminary step to account for reported gender differences in RA incidence by demonstrating that genetic polymorphisms within immune-related genes may have different effects in women and men, hence determining the way their immune systems respond to autoimmune stimuli. Nonetheless, further studies using independent populations are warranted to validate our findings.

## Supporting Information

Table S1 **Genotype frequencies and risk estimates of polymorphic loci in genes related to the macrophage/dendritic cell-induced immune response.** ^1^Models adjusted for age and gender. ^2^Models adjusted for age. ^3^ *p* value for testing of effect modification by gender was calculated utilizing an interaction term of gender and genetic polymorphism assuming a co-dominant model of inheritance. Results in bold show p<0.05. Abbreviations: OR, odds ratio; CI, confidence interval. All analyzed SNPs were in HWE in the control group with the exception of *Dectin-1*_rs16910631_ (p>0.01). This SNP was excluded from the analysis.
(DOCX)

Table S2 **Demographic and clinical characteristics of the RA population (Phase 2).** Data are means ± standard deviation. Abbreviations: RF, rheumatoid factor; Anti-CCP: anti-cyclic citrullinated peptide antibodies; DAS28, disease activity score; DMARDs, disease-modifying antirheumatic drugs. * Anti-CCP value was available only in 314 patients (254 women and 60 men).
(DOCX)

Table S3 **Demographic and clinical characteristics of the pooled population.** Data are means ± standard deviation. Abbreviations: RF, rheumatoid factor; Anti-CCP: anti-cyclic citrullinated peptide antibodies; DAS28, disease activity score; DMARDs, disease-modifying antirheumatic drugs. † Rheumatoid factor and anti-CCP values were available for 1.100 (907 women and 193 men) and 709 patients (582 women and 127 men), respectively. *Of those 1.212 RA patients (986 women and 226 men) were genotyped.
(DOCX)
