# Supplementary material for: Gender-Specific Effects of Genetic Variants within Th1 and Th17 Cell-Mediated Immune Response Genes on the Risk of Developing Rheumatoid Arthritis
Source: PLoS One. 2013 Aug 30;8(8):e72732. doi: 10.1371/journal.pone.0072732 (PMC3758336; doi:10.1371/journal.pone.0072732)
Supplement: Table S1 — Genotype frequencies and risk estimates of polymorphic loci in genes related to the macrophage/dendritic cell-induced immune response. 1Models adjusted for age and gender. 2Models adjusted for age. 3 p value for testing of effect modification by gender was calculated utilizing an interaction term of gender and genetic polymorphism assuming a co-dominant model of inheritance. Results in bold show p<0.05. Abbreviations: OR, odds ratio; CI, confidence interval. All analyzed SNPs were in HWE in the control group with the exception of Dectin-1 rs16910631 (p>0.01). This SNP was excluded from the analysis. (DOCX) [file pone.0072732.s001.docx]

**Table S1.** Genotype frequencies and risk estimates of polymorphic loci in genes related to the macrophage/dendritic cell-induced immune response.

|  | ***Overall*** | | |  | ***Men*** | | |  | ***Women*** | | |  |  |
| --- | --- | --- | --- | --- | --- | --- | --- | --- | --- | --- | --- | --- | --- |
| Variant information | Control (%) | Cases (%) | OR (95% CI)^1^ | *P value* | Control (%) | Cases (%) | OR (95% CI)^2^ | *P value* | Control (%) | Cases (%) | OR (95% CI)^2^ | *P value* | *P interaction^3^* |
|  |  |  |  |  |  |  |  |  |  |  |  |  |  |
| *DC-SIGN_*rs2287886 |  |  |  |  |  |  |  |  |  |  |  |  |  |
| G/G | 242 (49.0) | 199 (44.8) | 1.00 |  | 141 (49.8) | 38 (40.0) | 1.00 |  | 101 (47.9) | 161 (46.1) | 1.00 |  |  |
| A/G | 201 (40.7) | 191 (43.0) | 1.26 (0.93-1.70) |  | 119 (42.0) | 42 (44.2) | 1.52 (0.90-2.57) |  | 82 (38.9) | 149 (42.7) | 1.16 (0.80-1.69) |  |  |
| A/A | 51 (10.3) | 54 (12.2) | 1.20 (0.75-1.91) | 0.32 | 23 (8.1) | 15 (15.8) | **2.56 (1.16-5.63)** | **0.05** | 28 (13.3) | 39 (11.2) | 0.83 (0.48-1.45) | 0.47 | 0.071 |
| G/G vs. A/G+A/A | 252 (51.0) | 245 (55.2) | 1.24 (0.94-1.65) | 0.13 | 142 (50.2) | 57 (60.0) | **1.70 (1.03-2.78)** | **0.04** | 110 (52.1) | 188 (53.9) | 1.08 (0.76-1.53) | 0.67 |  |
|  |  |  |  |  |  |  |  |  |  |  |  |  |  |
| *DC-SIGN_*rs4804803 |  |  |  |  |  |  |  |  |  |  |  |  |  |
| A/A | 270 (54.2) | 285 (63.9) | 1.00 |  | 153 (53.7) | 66 (68.8) | 1.00 |  | 117 (54.9) | 219 (62.6) | 1.00 |  |  |
| A/G | 193 (38.8) | 135 (30.3) | **0.64 (0.47-0.86)** |  | 108 (37.9) | 27 (28.1) | 0.59 (0.34-1.01) |  | 85 (39.9) | 108 (30.9) | **0.67 (0.46-0.97)** |  |  |
| G/G | 35 (7.0) | 26 (5.8) | 0.79 (0.44-1.42) | **0.01** | 24 (8.4) | 3 (3.1) | 0.29 (0.08-1.04) | **0.03** | 11 (5.2) | 23 (6.6) | 1.23 (0.57-2.65) | 0.07 | 0.149 |
| A/A vs. A/G+G/G | 228 (45.8) | 161 (36.1) | **0.66 (0.49-0.88)** | **0.004** | 132 (46.3) | 30 (31.2) | **0.53 (0.32-0.89)** | **0.02** | 96 (45.1) | 131 (37.4) | 0.73 (0.51-1.04) | 0.08 |  |
|  |  |  |  |  |  |  |  |  |  |  |  |  |  |
| *DC-SIGN*_rs4804800 |  |  |  |  |  |  |  |  |  |  |  |  |  |
| A/A | 383 (75) | 351 (78.5) | 1.00 |  | 215 (73.1) | 75 (78.1) | 1.00 |  | 168 (77.4) | 276 (78.6) | 1.00 |  |  |
| A/G | 120 (23.5) | 87 (19.5) | 0.82 (0.59-1.16) |  | 74 (25.2) | 19 (19.8) | 0.72 (0.40-1.31) |  | 46 (21.2) | 68 (19.4) | 0.89 (0.58-1.36) |  |  |
| G/G | 8 (1.6) | 9 (2) | 1.40 (0.49-3.99) | 0.42 | 5 (1.7) | 2 (2.1) | 1.15 (0.20-6.64) | 0.54 | 3 (1.4) | 7 (2) | 1.54 (0.39-6.09) | 0.69 | 0.854 |
| A/A vs. A/G+G/G | 128 (25.1) | 96 (21.5) | 0.86 (0.62-1.20) | 0.37 | 79 (26.9) | 21 (21.9) | 0.75 (0.42-1.33) | 0.32 | 49 (22.6) | 75 (21.4) | 0.93 (0.61-1.40) | 0.72 |  |
|  |  |  |  |  |  |  |  |  |  |  |  |  |  |
| *DC-SIGN*_rs8112310 |  |  |  |  |  |  |  |  |  |  |  |  |  |
| T/T | 352 (71.1) | 318 (70.7) | 1.00 |  | 202 (71.1) | 72 (74.2) | 1.00 |  | 150 (71.1) | 246 (69.7) | 1.00 |  |  |
| A/T | 126 (25.4) | 114 (25.3) | 1.02 (0.73-1.41) |  | 70 (24.6) | 23 (23.7) | 1.05 (0.59-1.85) |  | 56 (26.5) | 91 (25.8) | 1.02 (0.69-1.52) |  |  |
| A/A | 17 (3.4) | 18 (4) | 1.20 (0.57-2.52) | 0.89 | 12 (4.2) | 2 (2.1) | 0.40 (0.08-1.90) | 0.43 | 5 (2.4) | 16 (4.5) | 2.07 (0.73-5.88) | 0.35 | 0.238 |
| T/T vs. A/T+A/A | 143 (28.9) | 132 (29.3) | 1.04 (0.76-1.42) | 0.81 | 82 (28.9) | 25 (25.8) | 0.93 (0.54-1.61) | 0.80 | 61 (28.9) | 107 (30.3) | 1.11 (0.76-1.62) | 0.60 |  |
|  |  |  |  |  |  |  |  |  |  |  |  |  |  |
| *DC-SIGN*_rs10410342 |  |  |  |  |  |  |  |  |  |  |  |  |  |
| G/G | 427 (87.7) | 396 (86.8) | 1.00 |  | 248 (87.6) | 85 (86.7) | 1.00 |  | 179 (87.8) | 311 (86.9) | 1.00 |  |  |
| C/G | 60 (12.3) | 55 (12.1) | 0.99 (0.64-1.52) |  | 35 (12.4) | 12 (12.2) | 0.89 (0.42-1.86) |  | 25 (12.2) | 43 (12.0) | 1.01 (0.59-1.73) |  |  |
| C/C | 0 (0.0) | 5 (1.1) | NA (0.00-NA) | **0.03** | 0 (0.0) | 1 (1.0) | NA (0.00-NA) | 0.12 | 0 (0.0) | 4 (1.1) | NA (0.00-NA) | 0.16 | 0.979 |
| G/G vs. C/G+C/C | 60 (12.3) | 60 (13.2) | 1.09 (0.71-1.66) | 0.70 | 35 (12.4) | 13 (13.3) | 0.99 (0.48-2.03) | 0.97 | 25 (12.2) | 47 (13.1) | 1.11 (0.66-1.89) | 0.69 |  |
|  |  |  |  |  |  |  |  |  |  |  |  |  |  |
| *DC-SIGN*_rs11465384 |  |  |  |  |  |  |  |  |  |  |  |  |  |
| C/C | 405 (81.2) | 377 (84.5) | 1.00 |  | 233 (81.5) | 85 (87.6) | 1.00 |  | 172 (80.8) | 292 (83.7) | 1.00 |  |  |
| C/T | 85 (17) | 63 (14.1) | 0.78 (0.53-1.14) |  | 47 (16.4) | 12 (12.4) | 0.68 (0.34-1.39) |  | 38 (17.8) | 51 (14.6) | 0.81 (0.51-1.30) |  |  |
| T/T | 9 (1.8) | 6 (1.4) | 0.64 (0.20-2.02) | 0.34 | 6 (2.1) | 0 (0) | 0.00 (0.00-NA) | 0.13 | 3 (1.4) | 6 (1.7) | 1.09 (0.27-4.43) | 0.68 | 0.930 |
| C/C vs. C/T+T/T | 94 (18.8) | 69 (15.5) | 0.76 (0.52-1.11) | 0.15 | 53 (18.5) | 12 (12.4) | 0.61 (0.30-1.24) | 0.16 | 41 (19.2) | 57 (16.3) | 0.83 (0.53-1.31) | 0.43 |  |
|  |  |  |  |  |  |  |  |  |  |  |  |  |  |
| *DC-SIGN*_rs11465413 |  |  |  |  |  |  |  |  |  |  |  |  |  |
| T/T | 406 (81.8) | 356 (80.2) | 1.00 |  | 233 (81.5) | 76 (81.7) | 1.00 |  | 173 (82.4) | 280 (79.8) | 1.00 |  |  |
| A/T | 90 (18.1) | 79 (17.8) | 1.05 (0.73-1.53) |  | 53 (18.5) | 15 (16.1) | 0.91 (0.47-1.77) |  | 37 (17.6) | 64 (18.2) | 1.13 (0.71-1.78) |  |  |
| A/A | 0 (0) | 9 (2) | NA (0.00-NA) | **7.00E-04** | 0 (0) | 2 (2.1) | NA (0.00-NA) | **0.01** | 0 (0) | 7 (2) | NA (0.00-NA) | **0.02** | 0.811 |
| T/T vs. A/T+A/A | 90 (18.1) | 88 (19.8) | 1.19 (0.83-1.71) | 0.35 | 53 (18.5) | 17 (18.3) | 1.04 (0.55-1.98) | 0.90 | 37 (17.6) | 71 (20.2) | 1.26 (0.80-1.97) | 0.31 |  |
|  |  |  |  |  |  |  |  |  |  |  |  |  |  |
| *DC-SIGN*_rs7252229 |  |  |  |  |  |  |  |  |  |  |  |  |  |
| G/G | 353 (69.9) | 315 (71) | 1.00 |  | 202 (69.9) | 71 (74) | 1.00 |  | 151 (69.9) | 244 (70.1) | 1.00 |  |  |
| G/C | 136 (26.9) | 115 (25.9) | 0.94 (0.69-1.30) |  | 76 (26.3) | 25 (26) | 0.92 (0.52-1.60) |  | 60 (27.8) | 90 (25.9) | 0.95 (0.64-1.40) |  |  |
| C/C | 16 (3.2) | 14 (3.1) | 0.97 (0.44-2.15) | 0.94 | 11 (3.8) | 0 (0) | 0.00 (0.00-NA) | **0.04** | 5 (2.3) | 14 (4) | 1.87 (0.65-5.35) | 0.44 | 0.941 |
| G/G vs. G/C+C/C | 152 (30.1) | 129 (29.1) | 0.95 (0.70-1.29) | 0.73 | 87 (30.1) | 25 (26) | 0.79 (0.46-1.37) | 0.40 | 65 (30.1) | 104 (29.9) | 1.02 (0.70-1.48) | 0.93 |  |
|  |  |  |  |  |  |  |  |  |  |  |  |  |  |
| *DC-SIGN*_rs7248637 |  |  |  |  |  |  |  |  |  |  |  |  |  |
| G/G | 390 (77.5) | 361 (80.6) | 1.00 |  | 222 (76.5) | 77 (80.2) | 1.00 |  | 168 (78.9) | 284 (80.7) | 1.00 |  |  |
| A/G | 108 (21.5) | 79 (17.6) | 0.82 (0.58-1.18) |  | 65 (22.4) | 18 (18.8) | 0.79 (0.43-1.45) |  | 43 (20.2) | 61 (17.3) | 0.84 (0.54-1.31) |  |  |
| A/A | 5 (1) | 8 (1.8) | 1.49 (0.44-5.11) | 0.44 | 3 (1) | 1 (1) | 0.82 (0.07-8.95) | 0.73 | 2 (0.9) | 7 (2) | 1.97 (0.40-9.76) | 0.49 | 0.850 |
| G/G vs. A/G+A/A | 113 (22.5) | 87 (19.4) | 0.86 (0.61-1.21) | 0.38 | 68 (23.4) | 19 (19.8) | 0.79 (0.43-1.43) | 0.43 | 45 (21.1) | 68 (19.3) | 0.89 (0.58-1.37) | 0.61 |  |
|  |  |  |  |  |  |  |  |  |  |  |  |  |  |
| *DC-SIGN*_rs11465421 |  |  |  |  |  |  |  |  |  |  |  |  |  |
| A/A | 164 (33.4) | 157 (35) | 1.00 |  | 96 (33.9) | 28 (29.2) | 1.00 |  | 68 (32.7) | 129 (36.5) | 1.00 |  |  |
| A/C | 239 (48.7) | 215 (47.9) | 1.00 (0.73-1.37) |  | 139 (49.1) | 52 (54.2) | 1.31 (0.75-2.27) |  | 100 (48.1) | 163 (46.2) | 0.86 (0.58-1.27) |  |  |
| C/C | 88 (17.9) | 77 (17.1) | 0.96 (0.64-1.45) | 0.98 | 48 (17) | 16 (16.7) | 1.22 (0.58-2.57) | 0.63 | 40 (19.2) | 61 (17.3) | 0.85 (0.51-1.40) | 0.71 | 0.468 |
| A/A vs. A/C+C/C | 327 (66.6) | 292 (65) | 0.99 (0.73-1.33) | 0.92 | 187 (66.1) | 68 (70.8) | 1.29 (0.76-2.18) | 0.35 | 140 (67.3) | 224 (63.5) | 0.86 (0.59-1.24) | 0.41 |  |
|  |  |  |  |  |  |  |  |  |  |  |  |  |  |
| *Dectin-1*_rs16910526 |  |  |  |  |  |  |  |  |  |  |  |  |  |
| A/A | 423 (85.1) | 380 (84.1) | 1.00 |  | 247 (85.5) | 82 (86.3) | 1.00 |  | 176 (84.6) | 298 (83.5) | 1.00 |  |  |
| A/C | 66 (13.3) | 65 (14.4) | 1.09 (0.73-1.64) |  | 37 (12.8) | 13 (13.7) | 0.98 (0.48-1.99) |  | 29 (13.9) | 52 (14.6) | 1.12 (0.68-1.84) |  |  |
| C/C | 8 (1.6) | 7 (1.6) | 0.81 (0.26-2.52) | 0.85 | 5 (1.7) | 0 (0) | 0.00 (0.00-NA) | 0.26 | 3 (1.4) | 7 (2) | 1.33 (0.33-5.30) | 0.85 | 0.957 |
| A/A vs. A/C+C/C | 74 (14.9) | 72 (15.9) | 1.06 (0.72-1.56) | 0.78 | 42 (14.5) | 13 (13.7) | 0.87 (0.43-1.74) | 0.69 | 32 (15.4) | 59 (16.5) | 1.14 (0.70-1.83) | 0.60 |  |
|  |  |  |  |  |  |  |  |  |  |  |  |  |  |
| *Dectin-1*_rs7309123 |  |  |  |  |  |  |  |  |  |  |  |  |  |
| C/C | 172 (34.5) | 146 (32.6) | 1.00 |  | 106 (36.8) | 34 (35.4) | 1.00 |  | 66 (31.3) | 112 (31.8) | 1.00 |  |  |
| C/G | 241 (48.3) | 223 (49.8) | 1.03 (0.75-1.41) |  | 135 (46.9) | 51 (53.1) | 1.18 (0.70-1.99) |  | 106 (50.2) | 172 (48.9) | 0.95 (0.64-1.41) |  |  |
| G/G | 86 (17.2) | 79 (17.6) | 1.01 (0.66-1.53) | 0.99 | 47 (16.3) | 11 (11.5) | 0.78 (0.35-1.74) | 0.54 | 39 (18.5) | 68 (19.3) | 1.09 (0.66-1.81) | 0.84 | 0.414 |
| C/C vs. C/G+G/G | 327 (65.5) | 302 (67.4) | 1.02 (0.76-1.38) | 0.89 | 182 (63.2) | 62 (64.6) | 1.08 (0.65-1.79) | 0.76 | 145 (68.7) | 240 (68.2) | 0.99 (0.68-1.43) | 0.94 |  |
|  |  |  |  |  |  |  |  |  |  |  |  |  |  |
| *Dectin-1*_rs3901533 |  |  |  |  |  |  |  |  |  |  |  |  |  |
| G/G | 301 (59.2) | 263 (59.8) | 1.00 |  | 176 (60.1) | 61 (63.5) | 1.00 |  | 125 (58.1) | 202 (58.7) | 1.00 |  |  |
| G/T | 173 (34.1) | 147 (33.4) | 0.95 (0.70-1.28) |  | 96 (32.8) | 32 (33.3) | 0.92 (0.55-1.55) |  | 77 (35.8) | 115 (33.4) | 0.95 (0.65-1.37) |  |  |
| T/T | 34 (6.7) | 30 (6.8) | 0.94 (0.54-1.66) | 0.93 | 21 (7.2) | 3 (3.1) | 0.42 (0.12-1.49) | 0.34 | 13 (6) | 27 (7.8) | 1.30 (0.64-2.64) | 0.69 | 0.286 |
| G/G vs. G/T+T/T | 207 (40.8) | 177 (40.2) | 0.95 (0.71-1.26) | 0.71 | 117 (39.9) | 35 (36.5) | 0.83 (0.50-1.37) | 0.47 | 90 (41.9) | 142 (41.3) | 1.00 (0.70-1.42) | 0.99 |  |
|  |  |  |  |  |  |  |  |  |  |  |  |  |  |
| *Dectin-1*_rs4763446 |  |  |  |  |  |  |  |  |  |  |  |  |  |
| T/T | 357 (72.1) | 318 (71) | 1.00 |  | 204 (71.8) | 71 (74) | 1.00 |  | 153 (72.5) | 247 (70.2) | 1.00 |  |  |
| C/T | 128 (25.9) | 117 (26.1) | 0.96 (0.70-1.33) |  | 74 (26.1) | 21 (21.9) | 0.77 (0.43-1.38) |  | 54 (25.6) | 96 (27.3) | 1.07 (0.72-1.59) |  |  |
| C/C | 10 (2) | 13 (2.9) | 1.77 (0.71-4.45) | 0.44 | 6 (2.1) | 4 (4.2) | 2.53 (0.67-9.65) | 0.25 | 4 (1.9) | 9 (2.6) | 1.44 (0.43-4.79) | 0.80 | 0.621 |
| T/T vs. C/T+C/C | 138 (27.9) | 130 (29) | 1.01 (0.74-1.39) | 0.93 | 80 (28.2) | 25 (26) | 0.88 (0.51-1.53) | 0.65 | 58 (27.5) | 105 (29.8) | 1.10 (0.75-1.61) | 0.64 |  |
|  |  |  |  |  |  |  |  |  |  |  |  |  |  |
| *Dectin-1*_rs7311598 |  |  |  |  |  |  |  |  |  |  |  |  |  |
| A/A | 357 (72.6) | 324 (71.7) | 1.00 |  | 196 (69.8) | 76 (77.5) | 1.00 |  | 161 (76.3) | 248 (70.1) | 1.00 |  |  |
| A/G | 119 (24.2) | 115 (25.4) | 1.09 (0.78-1.51) |  | 72 (25.6) | 21 (21.4) | 0.76 (0.42-1.36) |  | 47 (22.3) | 94 (26.6) | 1.31 (0.87-1.97) |  |  |
| G/G | 16 (3.2) | 13 (2.9) | 0.92 (0.39-2.13) | 0.86 | 13 (4.6) | 1 (1) | 0.24 (0.03-1.91) | 0.20 | 3 (1.4) | 12 (3.4) | 2.16 (0.60-7.85) | 0.22 | 0.075 |
| A/A vs. A/G+G/G | 135 (27.4) | 128 (28.3) | 1.07 (0.78-1.46) | 0.68 | 85 (30.2) | 22 (22.4) | 0.69 (0.39-1.20) | 0.18 | 50 (23.7) | 106 (29.9) | 1.36 (0.92-2.03) | 0.12 |  |
|  |  |  |  |  |  |  |  |  |  |  |  |  |  |
| *Dectin-2*_rs7134303 |  |  |  |  |  |  |  |  |  |  |  |  |  |
| A/A | 350 (69.9) | 287 (64.9) | 1.00 |  | 188 (65.7) | 70 (72.9) | 1.00 |  | 162 (75.3) | 217 (62.7) | 1.00 |  |  |
| A/G | 136 (27.1) | 138 (31.2) | 1.34 (0.98-1.84) |  | 88 (30.8) | 23 (24.0) | 0.72 (0.41-1.26) |  | 48 (22.3) | 115 (33.2) | **1.88 (1.26-2.81)** |  |  |
| G/G | 15 (3.0) | 17 (3.8) | 1.41 (0.65-3.09) | 0.15 | 10 (3.5) | 3 (3.1) | 0.71 (0.18-2.83) | 0.48 | 5 (2.3) | 14 (4.0) | 2.08 (0.72-5.97) | **0.004** | **0.011** |
| A/A vs. A/G+G/G | 151 (30.1) | 155 (35.1) | 1.35 (1.00-1.83) | **0.05** | 98 (34.3) | 26 (27.1) | 0.72 (0.42-1.23) | 0.22 | 53 (24.6) | 129 (37.3) | **1.90 (1.29-2.80)** | **9.00E-04** |  |
|  |  |  |  |  |  |  |  |  |  |  |  |  |  |
| *Dectin-2*_rs4264222 |  |  |  |  |  |  |  |  |  |  |  |  |  |
| C/C | 324 (64.3) | 254 (57.0) | 1.00 |  | 173 (59.9) | 60 (63.2) | 1.00 |  | 151 (70.2) | 194 (55.3) | 1.00 |  |  |
| C/T | 160 (31.8) | 165 (37.0) | **1.44 (1.06-1.95)** |  | 104 (36.0) | 31 (32.6) | 0.89 (0.53-1.50) |  | 56 (26.1) | 134 (38.2) | **1.90 (1.29-2.78)** |  |  |
| T/T | 20 (4.0) | 27 (6.0) | 1.67 (0.86-3.24) | **0.03** | 12 (4.2) | 4 (4.2) | 1.03 (0.31-3.45) | 0.91 | 8 (3.7) | 23 (6.5) | 2.19 (0.94-5.10) | **0.001** | **0.041** |
| C/C vs. C/T+T/T | 180 (35.7) | 192 (43.0) | **1.47 (1.10-1.96)** | **0.009** | 116 (40.1) | 35 (36.8) | 0.91 (0.55-1.49) | 0.70 | 64 (29.8) | 157 (44.7) | **1.93 (1.34-2.79)** | **3.00E-04** |  |
|  |  |  |  |  |  |  |  |  |  |  |  |  |  |
| *Dectin-2*_rs4459385 |  |  |  |  |  |  |  |  |  |  |  |  |  |
| C/C | 274 (55.2) | 264 (59.2) | 1.00 |  | 162 (56.2) | 58 (60.4) | 1.00 |  | 112 (53.9) | 206 (58.9) | 1.00 |  |  |
| C/T | 183 (36.9) | 149 (33.4) | 0.81 (0.60-1.10) |  | 106 (36.8) | 30 (31.2) | 0.90 (0.53-1.52) |  | 77 (37) | 119 (34) | 0.81 (0.55-1.17) |  |  |
| T/T | 39 (7.9) | 33 (7.4) | 0.83 (0.48-1.42) | 0.38 | 20 (6.9) | 8 (8.3) | 1.10 (0.44-2.76) | 0.88 | 19 (9.1) | 25 (7.1) | 0.71 (0.37-1.37) | 0.39 | 0.737 |
| C/C vs. C/T+T/T | 222 (44.8) | 182 (40.8) | 0.82 (0.61-1.09) | 0.17 | 126 (43.8) | 38 (39.6) | 0.93 (0.57-1.53) | 0.78 | 96 (46.1) | 144 (41.1) | 0.79 (0.55-1.12) | 0.19 |  |
|  |  |  |  |  |  |  |  |  |  |  |  |  |  |
| *MCP-1*_rs4586 |  |  |  |  |  |  |  |  |  |  |  |  |  |
| T/T | 191 (39.5) | 209 (46.4) | 1.00 |  | 121 (43.8) | 47 (48.0) | 1.00 |  | 70 (33.6) | 162 (46.0) | 1.00 |  |  |
| C/T | 227 (46.9) | 190 (42.2) | **0.68 (0.50-0.92)** |  | 117 (42.4) | 37 (37.8) | 0.82 (0.49-1.39) |  | 110 (52.9) | 153 (43.5) | **0.61 (0.42-0.89)** |  |  |
| C/C | 66 (13.6) | 51 (11.3) | 0.69 (0.44-1.09) | **0.03** | 38 (13.8) | 14 (14.3) | 0.98 (0.47-2.05) | 0.75 | 28 (13.5) | 37 (10.5) | **0.56 (0.31-0.99)** | **0.02** | 0.409 |
| T/T vs. C/T+C/C | 293 (60.5) | 241 (53.6) | **0.68 (0.51-0.91)** | **0.009** | 155 (56.2) | 51 (52.0) | 0.86 (0.53-1.40) | 0.55 | 138 (66.3) | 190 (54.0) | **0.60 (0.42-0.86)** | **0.005** |  |
|  |  |  |  |  |  |  |  |  |  |  |  |  |  |
| *MCP-1*_rs1024610 |  |  |  |  |  |  |  |  |  |  |  |  |  |
| A/A | 304 (61.2) | 262 (58.6) | 1.00 |  | 183 (63.8) | 58 (62.4) | 1.00 |  | 121 (57.6) | 204 (57.6) | 1.00 |  |  |
| T/A | 158 (31.8) | 157 (35.1) | 0.99 (0.73-1.34) |  | 82 (28.6) | 30 (32.3) | 1.02 (0.59-1.74) |  | 76 (36.2) | 127 (35.9) | 0.97 (0.67-1.40) |  |  |
| T/T | 35 (7) | 28 (6.3) | 0.89 (0.50-1.58) | 0.92 | 22 (7.7) | 5 (5.4) | 0.65 (0.23-1.83) | 0.68 | 13 (6.2) | 23 (6.5) | 1.04 (0.50-2.14) | 0.98 | 0.724 |
| A/A vs. T/A+T/T | 193 (38.8) | 185 (41.4) | 0.97 (0.73-1.30) | 0.85 | 104 (36.2) | 35 (37.6) | 0.94 (0.56-1.55) | 0.80 | 89 (42.4) | 150 (42.4) | 0.98 (0.69-1.39) | 0.90 |  |
|  |  |  |  |  |  |  |  |  |  |  |  |  |  |
| *MCP-1*_rs1024611 |  |  |  |  |  |  |  |  |  |  |  |  |  |
| A/A | 260 (54.5) | 275 (61.8) | 1.00 |  | 160 (58.4) | 60 (64.5) | 1.00 |  | 100 (49.3) | 215 (61.1) | 1.00 |  |  |
| G/A | 178 (37.3) | 139 (31.2) | **0.64 (0.47-0.88)** |  | 90 (32.9) | 24 (25.8) | 0.80 (0.46-1.42) |  | 88 (43.4) | 115 (32.7) | **0.60 (0.41-0.87)** |  |  |
| G/G | 39 (8.2) | 31 (7.0) | 0.76 (0.44-1.31) | **0.02** | 24 (8.8) | 9 (9.7) | 0.82 (0.35-1.93) | 0.71 | 15 (7.4) | 22 (6.2) | 0.68 (0.34-1.39) | **0.02** | 0.692 |
| A/A vs. G/A+G/G | 217 (45.5) | 170 (38.2) | **0.66 (0.50-0.89)** | **0.006** | 114 (41.6) | 33 (35.5) | 0.81 (0.48-1.35) | 0.41 | 103 (50.7) | 137 (38.9) | **0.61 (0.43-0.87)** | **0.006** |  |
|  |  |  |  |  |  |  |  |  |  |  |  |  |  |
| *MCP-1*_rs13900 |  |  |  |  |  |  |  |  |  |  |  |  |  |
| C/C | 271 (54.6) | 276 (60.9) | 1.00 |  | 164 (56.9) | 60 (61.2) | 1.00 |  | 107 (51.4) | 216 (60.9) | 1.00 |  |  |
| C/T | 190 (38.3) | 151 (33.3) | **0.71 (0.53-0.96)** |  | 101 (35.1) | 29 (29.6) | 0.87 (0.51-1.49) |  | 89 (42.8) | 122 (34.4) | **0.66 (0.46-0.95)** |  |  |
| T/T | 35 (7.1) | 26 (5.7) | 0.84 (0.47-1.49) | 0.08 | 23 (8.0) | 9 (9.2) | 0.90 (0.38-2.12) | 0.88 | 12 (5.8) | 17 (4.8) | 0.74 (0.34-1.63) | 0.08 | 0.704 |
| C/C vs. C/T+T/T | 225 (45.4) | 177 (39.1) | **0.73 (0.55-0.97)** | **0.03** | 124 (43.1) | 38 (38.8) | 0.88 (0.54-1.44) | 0.61 | 101 (48.6) | 139 (39.1) | **0.67 (0.47-0.95)** | **0.03** |  |
|  |  |  |  |  |  |  |  |  |  |  |  |  |  |
| *CCR2*_rs3918358 |  |  |  |  |  |  |  |  |  |  |  |  |  |
| A/A | 247 (49.7) | 214 (47.7) | 1.00 |  | 145 (50.7) | 44 (45.4) | 1.00 |  | 102 (48.3) | 170 (48.3) | 1.00 |  |  |
| A/C | 199 (40) | 174 (38.8) | 0.98 (0.72-1.32) |  | 110 (38.5) | 37 (38.1) | 1.14 (0.68-1.93) |  | 89 (42.2) | 137 (38.9) | 0.91 (0.63-1.32) |  |  |
| C/C | 51 (10.3) | 61 (13.6) | 1.50 (0.95-2.38) | 0.17 | 31 (10.8) | 16 (16.5) | 1.89 (0.91-3.96) | 0.25 | 20 (9.5) | 45 (12.8) | 1.34 (0.75-2.42) | 0.44 | 0.723 |
| A/A vs. A/C+C/C | 250 (50.3) | 235 (52.3) | 1.08 (0.82-1.43) | 0.59 | 141 (49.3) | 53 (54.6) | 1.30 (0.80-2.11) | 0.29 | 109 (51.7) | 182 (51.7) | 0.99 (0.70-1.40) | 0.96 |  |
|  |  |  |  |  |  |  |  |  |  |  |  |  |  |
| *CCR2*_rs743660 |  |  |  |  |  |  |  |  |  |  |  |  |  |
| G/G | 273 (56.2) | 264 (58.5) | 1.00 |  | 154 (55.6) | 52 (54.2) | 1.00 |  | 119 (56.9) | 212 (59.7) | 1.00 |  |  |
| A/G | 177 (36.4) | 147 (32.6) | 0.89 (0.65-1.20) |  | 102 (36.8) | 32 (33.3) | 0.92 (0.54-1.57) |  | 75 (35.9) | 115 (32.4) | 0.87 (0.60-1.26) |  |  |
| A/A | 36 (7.4) | 40 (8.9) | 1.30 (0.76-2.20) | 0.38 | 21 (7.6) | 12 (12.5) | 1.74 (0.76-3.97) | 0.36 | 15 (7.2) | 28 (7.9) | 1.06 (0.54-2.09) | 0.71 | 0.696 |
| G/G vs. A/G+A/A | 213 (43.8) | 187 (41.5) | 0.95 (0.72-1.27) | 0.75 | 123 (44.4) | 44 (45.8) | 1.05 (0.65-1.72) | 0.83 | 90 (43.1) | 143 (40.3) | 0.90 (0.63-1.28) | 0.55 |  |
|  |  |  |  |  |  |  |  |  |  |  |  |  |  |
| *CCR2*_rs1799864 |  |  |  |  |  |  |  |  |  |  |  |  |  |
| G/G | 405 (81.3) | 360 (80.9) | 1.00 |  | 236 (82.2) | 83 (87.4) | 1.00 |  | 169 (80.1) | 277 (79.1) | 1.00 |  |  |
| A/G | 84 (16.9) | 77 (17.3) | 0.86 (0.59-1.26) |  | 47 (16.4) | 11 (11.6) | 0.57 (0.27-1.19) |  | 37 (17.5) | 66 (18.9) | 1.02 (0.65-1.61) |  |  |
| A/A | 9 (1.8) | 8 (1.8) | 0.68 (0.24-1.91) | 0.59 | 4 (1.4) | 1 (1.1) | 0.54 (0.05-5.66) | 0.27 | 5 (2.4) | 7 (2) | 0.74 (0.23-2.38) | 0.87 | 0.531 |
| G/G vs. A/G+A/A | 93 (18.7) | 85 (19.1) | 0.84 (0.59-1.21) | 0.36 | 51 (17.8) | 12 (12.6) | 0.56 (0.28-1.15) | 0.10 | 42 (19.9) | 73 (20.9) | 0.99 (0.64-1.53) | 0.96 |  |
|  |  |  |  |  |  |  |  |  |  |  |  |  |  |

^1^Models adjusted for age and gender. ^2^Models adjusted for age. ^3^*p* value for testing of effect modification by gender was calculated utilizing an interaction term of gender and genetic polymorphism assuming a co-dominant model of inheritance. Results in bold show p<0.05. Abbreviations: OR, odds ratio; CI, confidence interval. All analyzed SNPs were in HWE in the control group with the exception of *Dectin-1*_rs16910631_ (p>0.01). This SNP was excluded from the analysis.
