# Supplementary material for: Gender-Specific Effects of Genetic Variants within Th1 and Th17 Cell-Mediated Immune Response Genes on the Risk of Developing Rheumatoid Arthritis
Source: PLoS One. 2013 Aug 30;8(8):e72732. doi: 10.1371/journal.pone.0072732 (PMC3758336; doi:10.1371/journal.pone.0072732)
Supplement: Table S2 — Demographic and clinical characteristics of the RA population (Phase 2). Data are means ± standard deviation. Abbreviations: RF, rheumatoid factor; Anti-CCP: anti-cyclic citrullinated peptide antibodies; DAS28, disease activity score; DMARDs, disease-modifying antirheumatic drugs. * Anti-CCP value was available only in 314 patients (254 women and 60 men). (DOCX) [file pone.0072732.s002.docx]

**Table S2.** Demographic and clinical characteristics of the RA population (Phase 2).

|  | ***RA patients*** | | |
| --- | --- | --- | --- |
|  | *Overall*  *(n=831)* | *Women (n=699)* | *Men (n=132)* |
| *Demographic characteristics* |  |  |  |
|  |  |  |  |
| *Age (years)* | 57.15 ± 12.85 | 56.67 ± 13.02 | 59.66 ± 11.66 |
|  |  |  |  |
| *Clinical assessment* |  |  |  |
|  |  |  |  |
| *Percentage of patients with RF positivity* | 75.98 | 75.35 | 79.41 |
| *Percentage of patients with positive anti-CCP ** | 77.72 | 75.61 | 88.06 |
| *Current DAS28 (average)* | 3.69 | 3.78 | 3.21 |
|  |  |  |  |
| *Treatments* |  |  |  |
|  |  |  |  |
| *DMARDs* |  |  |  |
| *Methotrexate (%)* | 657 (79.06) | 568 (81.26) | 89 (67.42) |
| *Leflunomide (%)* | 58 (6.98) | 44 (6.29) | 14 (10.61) |
| *Sulphasalazine (%)* | 153 (18.41) | 128 (18.31) | 25 (18.94) |
|  |  |  |  |
| *Biologic agents* |  |  |  |
| *Infliximab (%)* | 237 (28.52) | 201 (28.76) | 36 (27.27) |
| *Etanercept (%)* | 201 (24.19) | 168 (24.03) | 33 (25.00) |
| *Adalimumab (%)* | 98 (11.79) | 88 (12.59) | 10 (7.58) |
| *Abatacept (%)* | 12 (1.44) | 11 (1.57) | 1 (0.76) |
| *Rituximab (%)* | 60 (7.22) | 52 (7.44) | 8 (6.06) |
| *Tocilimumab (%)* | 53 (6.38) | 45 (6.44) | 8 (6.06) |
| *Others (%)* | 26 (3.13) | 24 (3.43) | 2 (1.51) |
|  |  |  |  |
| *Number of biologic agents* |  |  |  |
| *0* | 341 (41.03) | 287 (41.06) | 54 (40.91) |
| *1* | 353 (42.48) | 290 (41.49) | 63 (47.73) |
| *2* | 94 (11.31) | 82 (11.73) | 12 (9.09) |
| *3* | 30 (3.61) | 28 (4.01) | 2 (1.52) |
| *4* | 10 (1.20) | 10 (1.43) | 0 (0.00) |
| *>4* | 3 (0.36) | 2 (0.29) | 1 (0.76) |
|  |  |  |  |

Data are means ± standard deviation. Abbreviations: RF, rheumatoid factor; Anti-CCP: anti-cyclic citrullinated peptide antibodies; DAS28, disease activity score; DMARDs, disease-modifying antirheumatic drugs. *Anti-CCP value was available only in 314 patients (254 women and 60 men).
