# Supplementary material for: Gender-Specific Effects of Genetic Variants within Th1 and Th17 Cell-Mediated Immune Response Genes on the Risk of Developing Rheumatoid Arthritis
Source: PLoS One. 2013 Aug 30;8(8):e72732. doi: 10.1371/journal.pone.0072732 (PMC3758336; doi:10.1371/journal.pone.0072732)
Supplement: Table S3 — Demographic and clinical characteristics of the pooled population. Data are means ± standard deviation. Abbreviations: RF, rheumatoid factor; Anti-CCP: anti-cyclic citrullinated peptide antibodies; DAS28, disease activity score; DMARDs, disease-modifying antirheumatic drugs. † Rheumatoid factor and anti-CCP values were available for 1.100 (907 women and 193 men) and 709 patients (582 women and 127 men), respectively. *Of those 1.212 RA patients (986 women and 226 men) were genotyped. (DOCX) [file pone.0072732.s003.docx]

**Table S3.** Demographic and clinical characteristics of the pooled population.

|  | ***RA patients*** | | |
| --- | --- | --- | --- |
|  | *Overall*  *(n=1289)** | *Women (n=1059)* | *Men (n=230)* |
| *Demographic characteristics* |  |  |  |
|  |  |  |  |
| *Age (years)* | 57.64 ± 12.98 | 57.11 ± 13.04 | 60.07 ± 12.42 |
|  |  |  |  |
| *Clinical assessment* |  |  |  |
|  |  |  |  |
| *Percentage of patients with RF positivity* † | 74.09 | 73.43 | 77.20 |
| *Percentage of patients with positive anti-CCP* † | 74.61 | 73.88 | 77.95 |
| *Current DAS28 (average)* | 3.57 | 3.66 | 3.09 |
|  |  |  |  |
| *Treatments* |  |  |  |
|  |  |  |  |
| *DMARDs* |  |  |  |
| *Methotrexate (%)* | 916 (71.06) | 775 (73.18) | 141 (61.30) |
| *Leflunomide (%)* | 236 (18.31) | 176 (16.62) | 60 (26.09) |
| *Sulphasalazine (%)* | 193 (14.97) | 159 (15.01) | 34 (14.78) |
|  |  |  |  |
| *Biologic agents* |  |  |  |
| *Infliximab (%)* | 408 (31.65) | 342 (32.29) | 66 (28.70) |
| *Etanercept (%)* | 341 (26.45) | 284 (26.82) | 57 (24.78) |
| *Adalimumab (%)* | 222 (17.22) | 190 (17.94) | 32 (13.91) |
| *Abatacept (%)* | 55 (4.27) | 46 (4.34) | 9 (3.91) |
| *Rituximab (%)* | 172 (13.34) | 143 (13.50) | 29 (12.61) |
| *Tocilimumab (%)* | 73 (5.66) | 63 (5.95) | 10 (4.35) |
| *Others (%)* | 33 (2.56) | 30 (2.83) | 3 (1.30) |
|  |  |  |  |
| *Number of biologic agents* |  |  |  |
| *0* | 455 (35.30) | 363 (34.28) | 92 (40.00) |
| *1* | 548 (42.51) | 452 (42.68) | 96 (41.74) |
| *2* | 165 (12.80) | 138 (13.03) | 27 (11.74) |
| *3* | 76 (5.90) | 68 (6.42) | 8 (3.48) |
| *4* | 30 (2.33) | 27 (2.55) | 3 (1.30) |
| *>5* | 14 (1.09) | 10 (0.94) | 4 (1.74) |
|  |  |  |  |

Data are means ± standard deviation. Abbreviations: RF, rheumatoid factor; Anti-CCP: anti-cyclic citrullinated peptide antibodies; DAS28, disease activity score; DMARDs, disease-modifying antirheumatic drugs. † Rheumatoid factor and anti-CCP values were available for 1.100 (907 women and 193 men) and 709 patients (582 women and 127 men), respectively. *** Of those 1.212 RA patients (986 women and 226 men) were genotyped.
